# Display of Titles

**Published:** 1866-11

**Authors:** 


### DISPLAY OF TITLES.

EDITOR OF DENTAL REGISTER:—A TITLE appended to a name is ornamental, and so is a kink in a pig's tail. But for the pig to presume on the superiority of its swinehood, on account of the kink, or the man on that of his manhood on account of the title, is not proof of such superiority, but possibly of the reverse. When a man of many titles writes a book, the publisher, no doubt, regards the titles as part and parcel of the capital stock, and displays them accordingly. And even in this least objectionable mode, it appears to me that the matter is often disgustingly overdone.

But look at our professional journals, and at those of the medical profession. Isn't the display pretty strong when we learn on the title-page that the Journal is edited by JOHN HENRY NIGHTINGALE, D. D. S., M. D., A. M., Professor of Physiognomy in the Medical College of Wryface, Member of the Harmonic Society of Froghollow, etc., etc., and learn again at the head of an original article that the said John Nightingale has all these titles, and learn the same astounding facts in the table of contents calling attention to the said article? Of course John Henry has a *right* to use all these titles, and many more, from which it follows that it is none of my business whether he does or not; and from this it follows that I have said enough, and too much, and will, to relieve you of all responsibility, state that this is written by

DOCTOR GEORGE WATT, M. D., D. D. S., Professor of Chemistry and Therapeutics in the Ohio College of Dental Surgery.

LECTURER ON NATURAL SCIENCES IN THE UNION FEMALE SEMINARY OF XENIA, OHIO.

MEMBER OF THE MISSISSIPPI VALLEY ASSOCIATION OF DENTAL SURGEONS.

MEMBER OF THE OHIO DENTAL COLLEGE ASSOCIATION.

PRESIDENT OF THE OHIO STATE DENTAL SOCIETY.

SECOND PRESIDENT OF THE AMERICAN DENTAL ASSOCIATION.

FIRST PRESIDENT OF THE MAD RIVER VALLEY DENTAL SOCIETY.

CORRESPONDING MEMBER OF THE ODONTOGRAPHIC SOCIETY OF PENNSYLVANIA.

*** Honorary Member of the Young America Base-ball Club, Late Champion of the Cross-Roads Spelling School, etc., etc., etc., etc. SHOO!

